# Supplementary material for: “I’m not going to lay back and watch somebody die”: a qualitative study of how people who use drugs’ naloxone experiences are shaped by rural risk environment and overdose education/naloxone distribution intervention
Source: Harm Reduct J. 2023 Nov 10;20:166. doi: 10.1186/s12954-023-00900-z (PMC10636969; doi:10.1186/s12954-023-00900-z)
Supplement: Supplementary file 1 — Additional file 1. C2H OEND Materials. [file 12954_2023_900_MOESM1_ESM.pdf]

Good Samaritan & Naloxone Laws in Kentucky

"Good Samaritan Laws encourage bystanders to become *Good Samaritans* by summoning emergency responders without fear … of several legal consequences." Kentucky Harm Reduction Coalition

By state law in Kentucky (KY Rev Stat § 218A.133 (2015)), Good Samaritans who call 911 to report an overdose:

- ***CANNOT*** **be** ***charged*** **with drug possession**
- ***CANNOT*** **be** ***convicted*** **of drug possession**
- ***CANNOT*** **be** ***charged*** **with drug paraphernalia possession.**
- ***CANNOT*** **be** ***convicted*** **of drug paraphernalia drug possession**

PROVIDED THAT the following conditions are met:

1. The request for assistance is made "in good faith," meaning that it is a legitimate request for assistance and is not being made during a lawful search or arrest.
2. The assistance is sought for a drug overdose and is made to a public safety answering point (i.e. 911), law enforcement officer, or medical services practitioner;
3. The person who would otherwise be charged is the person who made the request, acts in concert with the requestor, or is the overdose victim;
4. The person remains with (or is) the victim until assistance is provided; and
5. The evidence for the potential charge or prosecution is obtained as result of the overdose and the need for assistance.

State law does *NOT* protect you from the following if you call 911 to report an overdose:

- **Being** ***arrested*** **for drug possession**
- **Being** ***arrested*** **for drug paraphernalia possession**
- **Being arrested, charged, or convicted for probation or parole violations**
- **Being arrest, charged, or convicted for other offenses**

By Kentucky law, a Good Samaritan who administers naloxone (also known as Narcan) to someone who may be overdosing is not criminally or civilly liable for any harms that may be caused by administering naloxone to the victim.

To reduce the risk of overdose:

- Take prescription opioids only as directed
- Do not mix opioids with other drugs or alcohol
- Make sure healthcare providers know everything you're taking, including prescription and over-the-counter medicines, vitamins, herbal supplements, alcohol, illegal drugs (e.g., heroin), and medications not prescribed to you

You may be at increased risk of opioid overdose:

- if you have not taken opioids for a period of time (reduced tolerance)
- if you have health problems such as asthma, breathing problems, liver or kidney problems, or are HIV-positive
- if you have experienced a previous overdose

Now that you have NARCAN®
Tell someone where it is and how to use it!

For locations to purchase naloxone visit:
http://KyStopOverdoses.ky.gov

To watch the How To Use Naloxone video visit:
https://CARE2HOPE.org/naloxone/

Or scan the QR code below:

Common opioids include:

GENERIC	BRAND NAME
**Hydrocodone**	Vicodin, Lorcet, Lortab, Norco, Zohydro
**Oxycodone**	Percocet, OxyContin, Roxicodone, Percodan
**Morphine**	MSContin, Kadian, Embeda, Avinza
**Codeine**	Tylenol with Codeine, TyCo, Tylenol #3
**Fentanyl**	Duragesic
**Hydromorphone**	Dilaudid
**Oxymorphone**	Opana
**Meperidine**	Demerol
**Methadone**	Dolophine, Methadose
**Buprenorphine**	Suboxone, Subutex, Zubsolv, Bunavail, Butrans

Heroin and carfentanil are also opioids

CARE2HOPE
University of Kentucky | Emory University

Opioid Overdose Recognition and Response Guide

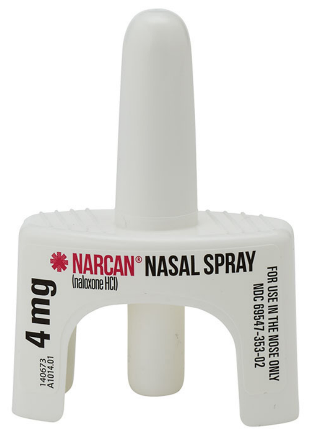

A GUIDE FOR PATIENTS, CAREGIVERS AND LOVED ONES

CARE2HOPE — Kentucky

Is it an overdose?

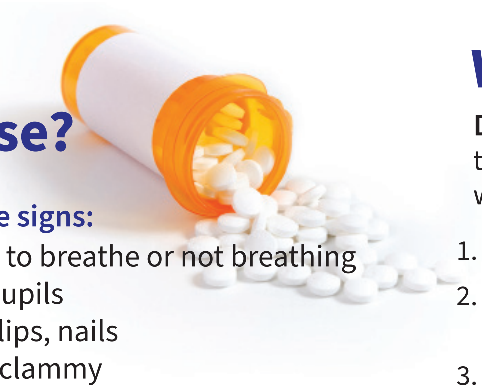

Look for these signs:

- Struggling to breathe or not breathing
- Pinpoint pupils
- Blue/gray lips, nails
- Skin pale, clammy
- Extreme sleepiness / inability to awaken verbally or by sternal rub

How to give NARCAN®:

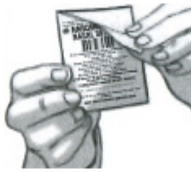

Peel

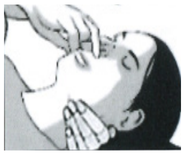

Place

Caution: do not activate (press) device until inserted into the nostril and you are ready to administer as all of the medication will be lost

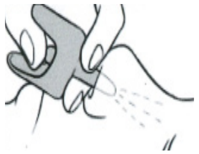

Press

NARCAN® Storage & Handling:

- Store NARCAN® at room temperature between 59°F to 77°F (or for *short* periods of time between 39°F to 104°F)
- Do not freeze NARCAN®
- Keep NARCAN® in its box until ready to use and protect from light
- Replace NARCAN® before the expiration date on the box

What do I do?

Don't panic! Try to wake the person by yelling their name and rubbing the middle of their chest with knuckles (sternal rub)

1. **Call 911**!
2. **Lay person on their back / begin rescue breathing** if breathing is stopped or very slow
3. **Peel back the tab** with the circle to open and remove NARCAN® from box.
4. Hold NARCAN® with your thumb on bottom of plunger and your first and middle fingers on either side of nozzle
5. Tilt person's head back; provide support under neck with your hand. **Gently insert tip of nozzle into one nostril** until your fingers on either side of nozzle are against the bottom of person's nose
6. **Press plunger firmly** to give dose of NARCAN®
7. Remove sprayer from nostril after giving dose
8. Continue rescue breathing if slow / no breathing
9. **IF AFTER 2-3** minutes person is still unresponsive with slow/no breathing repeat steps 3-8 above
10. **Roll person on side*** if breathing on own; continue to monitor breathing; begin rescue breathing again if required. **STAY with person until EMS arrives!**

***Putting someone in the recovery position will keep their airway clear and open, and ensures that any vomit or fluid won't cause them to choke.**

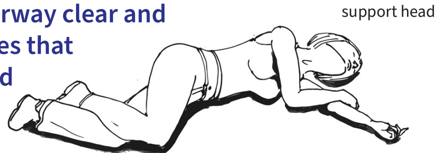

How to perform rescue breathing

Follow these steps for rescue breathing:

1. Place person on their back.
2. Tilt their chin up to open the airway.
3. Check to see if there is any object in their mouth blocking airway, such as gum, toothpick, undissolved pills, syringe cap, cheeked Fentanyl patch (these things have ALL been found in the mouths of overdosing people!). If so, remove object.
4. Plug their nose with one hand and give two even, regular-sized breaths. Blow enough air into their lungs to make their chest rise. (*Note*: the stomach/abdomen should NOT rise.) If you don't see their chest rise out of the corner of your eye, tilt the head back more and make sure you're plugging their nose.
5. Breathe again. Give one breath every 5 seconds.

NARCAN® Now

Mobile App Now Available

Important information in your hands

Access training and safety information in an easy-to-use app
