# Supplementary material for: “I’m not going to lay back and watch somebody die”: a qualitative study of how people who use drugs’ naloxone experiences are shaped by rural risk environment and overdose education/naloxone distribution intervention
Source: Harm Reduct J. 2023 Nov 10;20:166. doi: 10.1186/s12954-023-00900-z (PMC10636969; doi:10.1186/s12954-023-00900-z)
Supplement: Supplementary file 2 — Additional file 2. Codebook. [file 12954_2023_900_MOESM2_ESM.docx]

Supplementary File 2: Codebook

| **Name** | **Description** | **Example** |
| --- | --- | --- |
| **Barriers** | Refers to elements that prevented participants from achieving desired outcomes, before, during, or after C2H. Cross-code with environment sub-codes, e.g., employment/barriers, transportation/barriers | “Yeah. I mean, that, the transportation piece is... It's a huge obstacle when you have to get to work and if you don't have a way to do that, that makes it really hard.” |
| **Services** | Refers to programming participants accessed, before, during, or after C2H. Cross-code with environment sub-codes, e.g., housing/services, transportation/services | “I worked through Labor Ready, which was a daily work... A temp service.” |
| **Before C2H** | Refers to events, behaviors, and services accessed prior to participant joining C2H. Cross-code with relevant subject matter, e.g., housing/services/before C2H, criminal justice involvement/before C2H | “I started on meth back in 2016, and I would do it ... I started with one shot a day and then it got worse where I did it, shot up meth, about six times, eight times a day” |
| **During/after C2H** | Refers to events, behaviors, and services accessed after participant joined C2H. Cross-code with relevant subject matter, e.g., housing/services/during-after C2H, MAT/during-after C2H | “And then I am doing the IOP, intense outpatient. I'm doing it right now too.” |
| **Social Environment** | Refers to aspects of the participant’s social environment as part of the Risk Environment Framework (*see sub-codes)* | -- |
| *Stigma* | Refers to participant’s experienced judgement from others, particularly pertaining to drug use or criminal justice involvement | “I guess, it's how people are towards people on drugs and how people are that are on drugs to people. Their attitude and the way they look at people on both sides, really. People that don't do things, people that do do things. People are judgmental and really mean at times.” |
| *Family* | Refers to participant’s discussion of family members | “And every time I have a high craving or if I get too stressed out at the house I'm at, I'm here at my dad's house.” |
| *Friends* | Refers to participant’s discussion of non-family network members, including friends, former friends, and acquaintances. If highlighting lack of friends, cross-code with “barriers” | “Yeah, it's hard because I need somebody to teach me to actually drive. I need somebody that has time to do that. My family are all workers, and they work late hours and are tired when they come back in. But my friend on the other hand, he can teach me.” |
| *Romantic partner* | Refers to participant’s interactions with or perceptions of their spouse, girlfriend, boyfriend, or life partner | “We haven't even been married what, six months yet. We've lived together for the past two years though, and we hadn't been together five months when I got burned.” |
| *AA-NA* | Refers to participant’s experience or perception of alcoholics anonymous or narcotics anonymous support groups | “I want to do AA meetings, but there's no AA meetings here in this town.” |
| *Interpersonal conflict* | Refers to participant’s experience with arguing, fighting, or disagreeing with network members | “And then two months later, my mother and family coming down, and wanting to fight over stuff, and all that” |
| *Emotional support* | Refers to participant’s experience receiving social-emotional support (e.g., words of encouragement, validation, empathy, etc.) from REHN or other network members. If highlighting a lack of emotional support, cross-code with “barriers,” | “They definitely have been there. Even whenever I wasn't on this CARE2HOPE, they talked to me on my Messenger and stuff. And at one point I was going to go to treatment and, oh my God, they was all for it. They messaged every day, every day talking to me, they’re really, yeah, it helped me a lot. They helped me a lot.” |
| *Loss/grief* | Refers to participant’s experience having someone in their network die, and participant’s resulting emotions | “My best friend actually died of an overdose that we weren't able to bring him back from.” |
| **Economic Environment** | Refers to aspects of the participant’s economic environment as part of the Risk Environment Framework (*see sub-codes)* | -- |
| *Employment* | Refers to participant’s experience with jobs, or lack thereof. If highlighting lack of employment, cross-code with “barriers.” | “Yeah, the commute's the worst part. I actually like the job. I mean, I can relate to a lot of the people that I have to deal with on a daily basis.” |
| *Financial needs* | Refers to participant’s discussion of their funds | “I was broke. I needed 10 bucks too.” |
| **Physical Environment** | Refers to aspects of the participant’s physical environment as part of the Risk Environment Framework (*see sub-codes)* | -- |
| *Phone and internet access* | Refers to the impact that phone and internet access had on participant’s C2H intervention sessions or daily tasks | “Yeah. I know our internet, our signal here is bad in Frenchburg. Just in this rural area up here. Sometimes it goes out. You run into dead spots.” |
| *Housing* | Refers to participant’s experience with housing or lack thereof, including accessing housing services | “When I came home from prison, people don't want to rent to a convicted felon that's just coming out of prison.” |
| *Transportation* | Refers to participant’s experience with transportation or lack thereof, including experience accessing transportation services | “There's an NA group that meets up on the High Mountain, but I don't have transportation to get there right now.” |
| *Social services* | Refers to participant’s experience or perception of social services (e.g., SNAP, disability, CPS) | “Actually, I went into the food stamp office, and they had a flyer on the wall and I picked it up and I called” |
| *Emergency response systems* | Refers to ambulances, paramedics | *“And then when I woke up, there were paramedics around me and an old man with a dog. The paramedics were saying to me, when I came to, he said, "This man and this dog just saved your life."* |
| *“Rural”* | Participants’ inclusion of the word “rural” in their language | “Because in rural areas, ambulance doesn't always come.” |
| *Overdose* | Refers to participants description of overdose, either referring to themselves or to others | “I was really stuck with these people, and I would overdose and I would go to jail frequently, and that's the only time I would get sober. And then I would overdose, overdose, and overdose.” |
| *County drug use patterns/drug supply* | Refers to participant’s perceived changes or patterns in the drugs that people in their county use | “it was methamphetamine and marijuana and now everybody's on heroin and stuff.” |
| *SSP or harm reduction supplies* | Refers to syringe service programming or other harm reduction supplies | “You could go to Walmart and buy a 10 pack of syringes and you just had to sign a little list, but then they cut that out completely. So then it became kind of difficult to get a syringe.” |
| *Lack of services or inaccessible services* | Refers to participant’s inability to access needed services due to services being unavailable or otherwise inaccessible | “There's not too many resources here for myself.” |
| **Healthcare Environment** | Refers to aspects of the participant’s healthcare environment as part of the Risk Environment Framework (*see sub-codes)* | -- |
| *Doctor* | Refers to participant’s interactions with physician or other medical provider. Exclusion: paramedic | “It's also my family doctor. They're good people, so I've been knowing them a while.” |
| *Hospital/ICU* | Refers to participant’s experience with hospitals, emergency rooms, ICUs, or clinics | “All I remember is state police rushing me to the hospital. Then the next thing I know, I'm waking up in ICU in Hazard.” |
| *Non-drug health problems* | Refers to participant’s experience with health programs that are not definitively related to drug use | “I have thyroid disorder.” |
| *Drug-related health problems* | Refers to participant’s experience with health problems that are definitively related to drug use | “About using, and using dirty needles. I've had hepatitis C twice, I maybe have it again.” |
| *Mental health* | Refers to participant’s experience with mental health challenges, notably anxiety and depression | “Before moving, wow, mental health. Considerably. I was at a point to where I just... I didn't want to live anymore. And it was just such... I didn't have any hope. Just complete despair.” |
| *MAT* | Refers to participant’s experience or perceptions of medication-assisted treatment, including vivitrol, methadone, buprenorphine, naltrexone | “Vivitrol is a lot better than Suboxone. It's safer and it helps you stay off meth and opiates at the same time.” |
| *Drug treatment* | Refers to participant’s experiences with or perceptions of substance use treatment, including residential, outpatient, and intensive outpatient programming | “I went to rehab and I was in a career that kind of don't want you to do things like that” |
| **Law Enforcement Environment** | Refers to aspects of the participant’s law enforcement environment as part of the Risk Environment Framework (*see sub-codes)* | -- |
| *Police/911* | Refers to participant’s interactions with police officers or experience calling 911 | “To know the supreme court issued it, deemed it to be legal, I guess. I've heard police officers say that it's not against the law, and correctional officers.” |
| *Probation/parole* | Refers to participant’s experiences with and perceptions of probation and/or parole | “I do have a parole officer. I'm still on parole with him.” |
| *Criminal justice involvement* | Inclusion: jail, prison, reentry, criminal record | “if you have anything drug related on your record, they won't help you” |
| **Political Environment** | Refers to aspects of the participant’s political environment as part of the Risk Environment Framework (*see sub-codes)* | -- |
| Good Samaritan Laws | Refers to participant knowledge and perception of laws that exempt individuals from persecution in aftermath of overdose | “Yeah. A lot of people, it kind of stunned them because there was like, "Whoa, there's actually..." And with the Good Samaritan law, I didn't know anything about that until REHN told me about it.” |
| **Drugs used** | Refers to substances used or described by participant | “I guess they were cutting it with fentanyl because they had two different baggies, one with the meth in it and one with the fentanyl in it. They had made him a bag, but nothing with fentanyl because they were so messed up.” |
| *Heroin* | Refers to participant’s use or perception of heroin | “To me, I feel like heroin and methamphetamines have pretty much, I think, took over pretty much. About everybody you see, just about, is on it.” |
| *Fentanyl* | Refers to participant’s use or perception of fentanyl | “And then the fact that they was giving me stuff to help me to where I wouldn't die with that fentanyl, that helped too, because I was using bad the first time I came” |
| *Prescription opiates* | Refers to participant’s use or perception of prescription opiates | “He gave me 16 Norco 5s. The prescription is laying here on my safe to this day.” |
| *Methamphetamine* | Refers to participant’s use or perception of methamphetamine | “I turned to meth and I hid and I ran..” |
| *Alcohol* | Refers to participant’s use or perception of alcohol | “Well, she gave me a pill and I drank some alcohol. I don't know what kind of pill it was, but I blacked out.” |
| *Marijuana* | Refers to participant’s use or perception of marijuana | “I don't care what someone else tells me, you could get addicted on pot. You could smoke pot all day long all your life” |
| *Cocaine* | Refers to participant’s use or perception of cocaine | “Yes. When alcohol and marijuana was around, it wasn't that big of a deal, but then when this meth and cocaine and heroin and all that come into play, it's really changed things.” |
| **Drug use mechanism** | Refers to the route(s) of administration through which participants use drugs | -- |
| *Injection* | Refers to participant experience or perception of injecting drugs using a syringe | “Before, I would've maybe used if I didn't have a clean needle, I might used somebody else's.” |
| *Smoking* | Refers to participant experience or perception of smoking | “my friends that was already smoking, you know, and I was like ... One puff, one puff is all it takes. One puff of a cigarette.” |
| *Snorting* | Refers to participant’s experience or perception of snorting drugs | “I am a drug user and I don't inject, but I do it through my nose.” |
| *Swallowing pills* | Refers to participant’s experience of perception of swallowing pills | “Really painful, because I don't take the pain pills. I could only take five, so it was better than I thought.” |
| **Drug use frequency** | Refers to how often participant reports using drugs | “No, I don't use anything. I don't use anything. And plus where I do drug test it says I don't. Like I said, I do this for previous use, to help with it” |
| *Injection frequency* | Refers to how often participant reports injecting drugs, including discussion of changes in frequency | “I don't shoot up. I never did shoot up. A lot of my friends do.” |
| **Drug cessation** | Refers to participant’s experience stopping or reducing drug use, including discussions of withdrawal, craving, and difficulty quitting | “Because when you're coming off heroin, I mean, it's very hard, especially the way I've done it, because I just quit it cold turkey.” |
| *relapse* | Refers to participant’s experience or perception of the resumption of substance use after a period of abstinence | “Because of expectations always turned into resentment, and resentment always returned into vengeance, and vengeance always turned into a relapse For me.” |
| *craving* | Refers to participant’s experience with intense urge to use drugs | “Vivitrol is a lot better than Suboxone. It's safer and it helps you stay off meth and opiates at the same time. You don't have that craving. And it also helps you not have cravings for smoking cigarettes or smoking weed.” |
| *withdrawal* | Refers to participant’s experience or perception of physiological withdrawal symptoms following a period of drug cessation | “So I decided to go through the withdrawal from my pain meds. Wasn't as bad with the Suboxone.” |
| **Joining C2H** | Refers to participant’s experience learning about and deciding to join the C2H intervention | “I heard it from the health department, my probation office. And I also found it on Facebook and I called the number and they called me back.” |
| **C2H intervention experience** | Refers to participant’s experience engaging in the C2H intervention | -- |
| ***REHN*** | Refers to participant’s description of working with REHN | “[REHN] just helped me get there. And he helped me finish it, I think, because I don't want him to know that part of me or know nothing about that part of me.” |
| *REHN relationship* | Refers to the participant’s social/personal relationship to their REHN. If highlighting a lack of relationship, cross-code with “barriers” | “*They [rehn] was very understanding of the things. I felt comfortable with them.. It was more of a friendship. We had a bond, a familiar bond.”* |
| *REHN lived experience* | Refers to participant’s description of REHN lived experience, including experience with substance use and/or living in rural community | “And they [rehn]'s local too. They [rehn] lives local and so they [rehn]'s familiar with my area and the lifestyle that everyone lives here.” |
| ***Goal setting and attainment*** | Refers to participant’s experience setting and working towards goals in C2H intervention | “My goals are to get my license back, my GED, and to take college courses.” |
| ***Education/Informational Support*** | Refers to participant learning new information/skills from C2H staff: Inclusion: had never heard of Narcan/FTS, didn’t know how to use Narcan/FTS | “And I learned a lot about STDs talking through that program, through the CARE2HOPE program that I wasn't aware of, and I've got a medical background.” |
| *Receiving* ***fentanyl test strips*** | Refers to participant’s experience receiving fentanyl test strips from C2H staff | “I had never seen them. I had heard people talk about them, but I had never actually seen them, seen them, until [REHN] had gave me some of those” |
| *Utilizing fentanyl test strips* | Refers to participant’s experience utilizing C2H fentanyl test strips | “Yeah, I'll pack them everywhere. Because you never know when somebody else, you're going to have... Somebody's going to need to test it to see what the results are.” |
| *FTS perceptions/feelings* | Refers to participant’s feelings, opinions, and perceptions of fentanyl test strips | “Saved a lot of people's lives. It has actually cut down on some people that we know. We know a lot of people. We have lost a lot of people because of fentanyl, but now that them come into the picture, it kind of slowed down, should I say?” |
| *Receiving* ***Narcan*** | Refers to participant’s experience receiving Narcan from C2H staff | “It really helped me, talking to [them]. [They] gave me Narcan.” |
| *Carrying Narcan* | Refers to participants’ storage of Narcan including whether they opt to carry Narcan on their person | “I haven't had to use it yet. It's in the cabinet at the house that I usually stay at. I can't use it because I'm allergic to it.” |
| *Utilizing Narcan* | Refers to participant’s experience utilizing Narcan. If highlighting non-C2H Narcan, cross-code with “before C2H” | “I would have lost those two a couple of times because they were[...] Just a squirt to get them to wake up, so that video that I watched up there probably saved their lives.” |
| *Narcan used on participant* | Refers to participant’s experience having Narcan used on them | “It wasn't that I was upset that I was being resuscitated. It was the feeling that the Narcan gave me. It made my whole body go ice cold, and I started shaking. Because Narcan reverses the effects of the heroin, which made you go in sudden, rapid withdrawal times 50.” |
| *Narcan perceptions/feelings* | Refers to participant’s feelings, opinions, and perceptions of Narcan | “Even if you don't need it, it's a good, people see it as a good thing to have.” |
| *Narcan knowledge, existence* | Refers to participant’s prior knowledge or learning of what Narcan is | “I didn’t know what it was” |
| *Narcan knowledge, effectiveness* | Refers to participant’s prior knowledge or learning of Narcan’s effectiveness in reversing overdose | “At first I was like… ain’t no way it saves somebody’s life. Yeah, it does. It works good” |
| *Narcan knowledge, amnesty* | Refers to participant’s knowledge or learning of Good Samaritan or medical amnesty laws/policies | “you can be arrested, you can be charged, but they can’t prosecute you and make it stick if you’re having Narcan in your pocket.” |
| *Narcan, social obligation* | Refers to participant’s self-imposed social role as a community helper, regarding Narcan | *“I can’t walk by somebody laying on the ground and not try to help… That’s somebody’s daddy or mother or daughter or son… I won’t walk by.”* |
| *Narcan, “safer”* | Refers to participant feeling “safer with Narcan” than without it | “I feel a lot safer with Narcan” |
| *Narcan, unpredictable nature of overdose* | Refers to participants perception of community overdose as unpredictable or chaotic, regarding Narcan | “I know when I have [Narcan] on me, because you can't ever tell in wherever you're at, what kind of situations going on, anything can happen in the spur of a moment. And I know as long as I've got that on me, if something like that ever happens around me, it could save somebody's life.” |
| *Narcan, stigma* | Refers to participants perceived stigma from law enforcement and/or community members regarding Narcan | “if I’m carrying Narcan, then that’s going to make [police] judge me or question me more and wonder why I have that.” |
| *Narcan, recipient reaction* | Refers to participant recounting or anticipating instances a person’s physiological or emotional reaction to receiving Narcan | “He says that you feel rough after you get Narcan…for a day or two.” |
| *Narcan, loss of high* | Refers specifically to a Narcan recipient being frustrated that they can no longer feel effect of drugs | “They didn’t want their high to go away. They were so high that it could kill them, but they didn’t want to lose their high.” |
| *Narcan, economic loss* | Refers specifically to Narcan recipient being frustrated that they spent limited funds on drugs they can no longer feel the effect of | “You don’t want to lose that feeling that you paid for” |
| *Narcan, prior communication* | Refers to participant recounting conversations they have had with network members regarding Narcan, prior to overdose events | “’If you nod out, and if you don’t respond to me… I will Narcan you.’ Even before they [use drugs], I’m like, ‘I do have Narcan. I will Narcan you.’” |
| ***HCV testing****, feelings before* | Refers to participant’s emotions and thoughts prior to participating in C2H HCV testing | “Actually, I was kind of nervous because when you're an IV user, you can give it to yourself and I've always been clean. I've never carried no needles, never used any needles after anybody else but you can get it yourself.” |
| *HCV testing, experience* | Refers to participant’s experience participating in C2H HCV testing | “She asked me if it would be okay for her to test me, and I had been tested at the Health Department before. The HIV test I had, but not the Hep C test I had never been. So she told me what she was going to do to, I said okay.” |
| *HCV testing, result* | Refers to participant learning of their HCV test result | “It was good. I was happy to find out that I am negative. I had hepatitis, but my liver supposedly cleared itself” |
| *HCV testing, aftermath* | Refers to participant experience after learning their HCV result, including decision to engage with HCV treatment and behavior change | “I've not even got started in [the Hep C treatment] yet. I'm actually going to be doing that today after I get out of here. |
| ***HIV testing****, feelings before* | Refers to participant’s emotions and thoughts prior to participating in C2H HIV testing | “Nerve-racking. I don't know. It was very nerve-racking.” |
| *HIV testing, experience* | Refers to participant’s experience participating in C2H HIV testing | “I had come in to the office, and I done that, I done the HIV testing and I also done the hep C testing.” |
| *HIV testing, result* | Refers to participant learning of their HIV test result | “[Learning my test result felt] relieving because I knew I was negative.” |
| *HIV testing, aftermath* | Refers to participant experience after learning their HIV result, including decision to engage with HIV treatment and behavior change | “It went wonderful because I didn't have HIV, tell you that. Because I was nervous. I ain't going to lie because my partner is not so faithful as I am.” |
| ***Referral to non-C2H services*** | Refers to participant’s experience being connected to external services through their REHN | “He connected me with R and RJ. He helped me get my food stamps. He helped me do it all, [REHN] did.” |
| *Non-C2H staff treatment* | Refers to participant’s experience engaging with non-C2H staff | “They really been better than me than my own family have.” |
| *REHN role in referral* | Refers to participant’s recounting of how REHN made linkage (e.g., providing participant with phone number, accompanying participant to appointment, etc.) | “They went pretty much to all lengths to make sure, if they didn't know how to find information out for me or to help me with the situations that I was having, they always knew who to call or where to see me to, and stuff like that. They helped. They went to all lengths to help” |
| *Not connected to services* | Refers to participant’s discussion of not being connected to any services, including reasons they were not connected to services | “There's not really much [REHN] can do because the things I need to do is getting the money for [my goals] being able to have the money to buy the car. There's not much that no one else can really do to help me, unless they say, ‘Oh, I believe in you.’ That's about all they can do.” |
| ***Recommendations*** | Refers to ideas that participant has to improve the C2H intervention | “So just keep your people within your region. Because like I said, if you did expand it to different parts of the state or even the western part of Kentucky is different than the eastern part. So just be mindful of your local people. Don't try to send a city person to help a hillbilly.” |
| ***Unmet needs*** | Refers to participant needs that were not adequately met by C2H intervention | “I just wish I could get those test strips, fentanyl. If somebody might have a question about drugs, I could let them know I had those if they needed them.” |
